# Phosphofructo-1-Kinase Deficiency Leads to a Severe Cardiac and Hematological Disorder in Addition to Skeletal Muscle Glycogenosis

**DOI:** 10.1371/journal.pgen.1000615

**Published:** 2009-08-21

**Authors:** Miguel García, Anna Pujol, Albert Ruzo, Efrén Riu, Jesús Ruberte, Anna Arbós, Anna Serafín, Beatriz Albella, Juan Emilio Felíu, Fátima Bosch

**Affiliations:** 1 Center of Animal Biotechnology and Gene Therapy, Universitat Autònoma de Barcelona, Bellaterra, Barcelona, Spain; 2 Department of Biochemistry and Molecular Biology, School of Veterinary Medicine, Universitat Autònoma de Barcelona, Bellaterra, Barcelona, Spain; 3 CIBER de Diabetes y Enfermedades Metabólicas Asociadas (CIBERDEM), Barcelona, Spain; 4 Department of Animal Health and Anatomy, School of Veterinary Medicine, Universitat Autònoma de Barcelona, Bellaterra, Barcelona, Spain; 5 Hematopoiesis and Gene Therapy Division, CIEMAT, Madrid, Spain; University of Washington, United States of America

## Abstract

Mutations in the gene for muscle phosphofructo-1-kinase (*PFKM*), a key regulatory enzyme of glycolysis, cause Type VII glycogen storage disease (GSDVII). Clinical manifestations of the disease span from the severe infantile form, leading to death during childhood, to the classical form, which presents mainly with exercise intolerance. PFKM deficiency is considered as a skeletal muscle glycogenosis, but the relative contribution of altered glucose metabolism in other tissues to the pathogenesis of the disease is not fully understood. To elucidate this issue, we have generated mice deficient for PFKM (*Pfkm^−/−^*). Here, we show that *Pfkm^−/−^* mice had high lethality around weaning and reduced lifespan, because of the metabolic alterations. In skeletal muscle, including respiratory muscles, the lack of PFK activity blocked glycolysis and resulted in considerable glycogen storage and low ATP content. Although erythrocytes of *Pfkm^−/−^* mice preserved 50% of PFK activity, they showed strong reduction of 2,3-biphosphoglycerate concentrations and hemolysis, which was associated with compensatory reticulocytosis and splenomegaly. As a consequence of these haematological alterations, and of reduced PFK activity in the heart, *Pfkm^−/−^* mice developed cardiac hypertrophy with age. Taken together, these alterations resulted in muscle hypoxia and hypervascularization, impaired oxidative metabolism, fiber necrosis, and exercise intolerance. These results indicate that, in GSDVII, marked alterations in muscle bioenergetics and erythrocyte metabolism interact to produce a complex systemic disorder. Therefore, GSDVII is not simply a muscle glycogenosis, and *Pfkm^−/−^* mice constitute a unique model of GSDVII which may be useful for the design and assessment of new therapies.

## Author Summary

Type VII glycogen storage disease (GSDVII), or Tarui disease, is a rare genetic disorder characterized by glycogen accumulation in skeletal muscle. The molecular cause is loss of activity of the muscle isoform of phosphofructokinase (PFK), which phosphorylates fructose-6-phosphate to fructose-1,6-bisphosphate, commiting glucose to glycolysis. Entry of fructose-6-phosphate into glycolysis is thus blocked, increasing glycogen synthesis and accumulation. Clinical manifestations of the disease are heterogeneous, ranging from exercise intolerance to early childhood death. To further understand the human pathology, we generated mice lacking muscle PFK. As in human patients, these mice showed severe exercise intolerance, hemolysis, and most died young. Lack of glycolysis in skeletal muscle also causes alterations in bioenergetics and compensatory changes in key metabolic genes. Additionally, although erythrocytes retained 50% of normal PFK activity, their overall functionality was impaired, aggravating the muscle dysfunction. Moreover, marked metabolic alterations in the heart lead to chronic hypertrophy, suggesting that cardiac pathology in GSDVII may be underestimated or misdiagnosed. This study indicates that this disease is more complex than a muscle glycogenosis and that symptoms other than those classically described should be taken into consideration. Finally, this animal model will enable us to develop new therapeutic approaches and better diagnostic tools.

## Introduction

Phosphofructo-1-kinase (PFK) is a tetrameric enzyme that phosphorylates fructose-6-phosphate to fructose-1,6-bisphosphate, committing glucose to glycolysis. Three PFK isoenzymes, encoded by separate genes, have been identified in mammals: muscle-type (*PFKM*), liver-type (*PFKL*), and platelet-type (*PFKP*), all of which are expressed in a tissue specific manner [1]. Thus, skeletal muscle expresses only PFKM homotetramers, liver mainly PFKL homotetramers, although it can also express M- and P-type subunits, while erythrocytes contain PFKM and PFKL heterotetramers [2],[3]. Several mutations in *PFKM* cause type VII glycogen storage disease (GSDVII), which is a rare disease described by Tarui (Tarui's disease) [4]. GSDVII is inherited as an autosomal recessive trait and patients show loss of PFK activity in skeletal muscle and also partial deficiency in erythrocytes. Although GSDVII is characterized by accumulation of glycogen in skeletal muscle and hemolysis, there are several subtypes with different clinical features. No genotype-phenotype correlation explaining the phenotypic heterogeneity of the disease has been described [5]. It can be detected as a severe form with onset in infancy with hypotonia, limb weakness, progressive myopathy and respiratory failure leading to death early in the childhood [6],[7]. Neonatal mortality may be responsible for the low number of cases diagnosed. Adult patients with the classical form of the disease develop myopathy with muscle cramps and myoglobinuria when exercised as well as compensated haemolytic anemia.

GSDVII is considered as a muscle glycogenosis. Although, alterations in oxidative metabolism and bioenergetics in skeletal muscle have also been described in human patients, few data on metabolic and fiber structural changes are available. In addition, the contribution of altered glucose metabolism in other tissues to the pathogenesis of the disease is not fully understood and may also lead to misdiagnosis [8]. No therapies are available for GSDVII patients and development of effective treatments requires both understanding the molecular mechanisms that lead to the disease and the development of animal models in which to test new treatments. Inherited PFKM deficiency has only been described in dogs [9],[10]. However, PFKM deficient dogs exhibit mild muscle disease not closely reproducing the human muscle pathology [11]. In the present study, to determine the molecular mechanisms underlying this disease, we have generated mice lacking the muscle isoform of PFK. We found that PFKM deficiency leads to marked alterations in muscle bioenergetics and erythrocyte metabolism that interact to produce the complex pathology characteristic of GSDVII. The availability of the *Pfkm* ^−/−^ mouse model allows the study of GSDVII as a systemic disorder, not simply as muscle glycogenosis.

## Results

### *Pfkm^−/−^* mice exhibit high lethality and skeletal muscle glycogenosis

**Figure 1:**
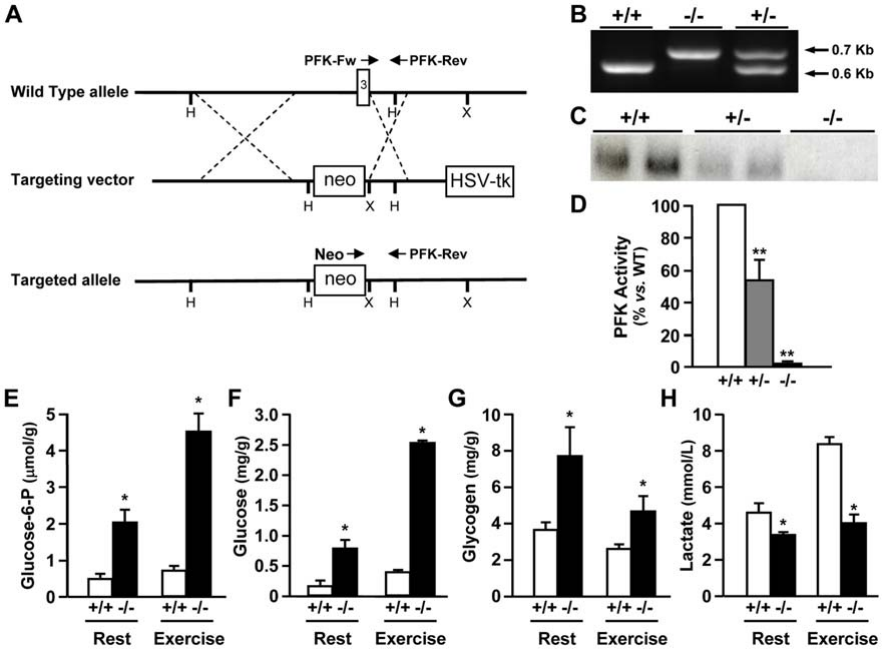
Generation of *Pfkm^−/−^* mice and the effect of *Pfkm* ablation on skeletal muscle glucose metabolism. (A) Schematic representation of the wild-type *Pfkm* locus (top), targeting vector (middle) and targeted allele (bottom). The positions of *Hind*III (H) and *Xba*I (X) cleavage sites, the *neo* ^R^ (neo) and herpes simplex virus thymidine kinase (*HSV-tk*) genes, and the location of PCR primers used to detect wild-type (PFK-Fw and PFK-Rev) and targeted (Neo and PFK-Rev) alleles are shown. (B) PCR analysis of DNA from wild type (*+/+*), *Pfkm^+/−^* (*+/−*) and *Pfkm^−/−^* (−/−) mice using the primers shown in (A). The 0.6 Kb band corresponds to the wild-type allele and the 0.7 Kb band to the mutant allele. (C) Expression of *Pfkm* in skeletal muscle. Total RNA was obtained from gastrocnemius muscle and analyzed by Northern blot. A representative Northern blot hybridized with a *Pfkm* probe is shown. (D) PFK activity was determined in skeletal muscle as indicated in Materials and Methods. Basal PFK activity in wild-type mice was 36±2.4 U/g tissue. (E–G) Glucose-6-phosphate (E), glucose (F) and glycogen (G) concentrations were determined in perchloric extracts of skeletal muscle from 2–3 month-old wild-type (+/+) and and *Pfkm^−/−^* (−/−) mice, in rest and after exercise (5 min), as indicated in Materials and Methods. (H) Serum lactate levels in wild-type (+/+) and *Pfkm^−/−^* (−/−) mice, in rest and after exercise (5 min). Results in D-H are mean±SEM of five to eight mice per group. *P<0.05, **P<0.01 *vs.* wild-type.

To generate *Pfkm* deficient mice, standard gene-targeting methods in mouse embryonic stem cells were used. Homologous recombination of the targeting construct resulted in the deletion of the 5′ promoter region and exon 3, which contains the translation start codon (Figure 1A). The presence of heterozygous and homozygous (*Pfkm^+/−^* and *Pfkm^−/−^*) mice was confirmed by Southern blot (data not shown) and by PCR (Figure 1B). *Pfkm^+/−^* mice were viable and fertile while *Pfkm*-null mice presented high lethality around weaning (about 60%) and those surviving died early during adulthood, at around 3 to 6 month of age, although few animals survived for more than one year.

**Figure 2:**
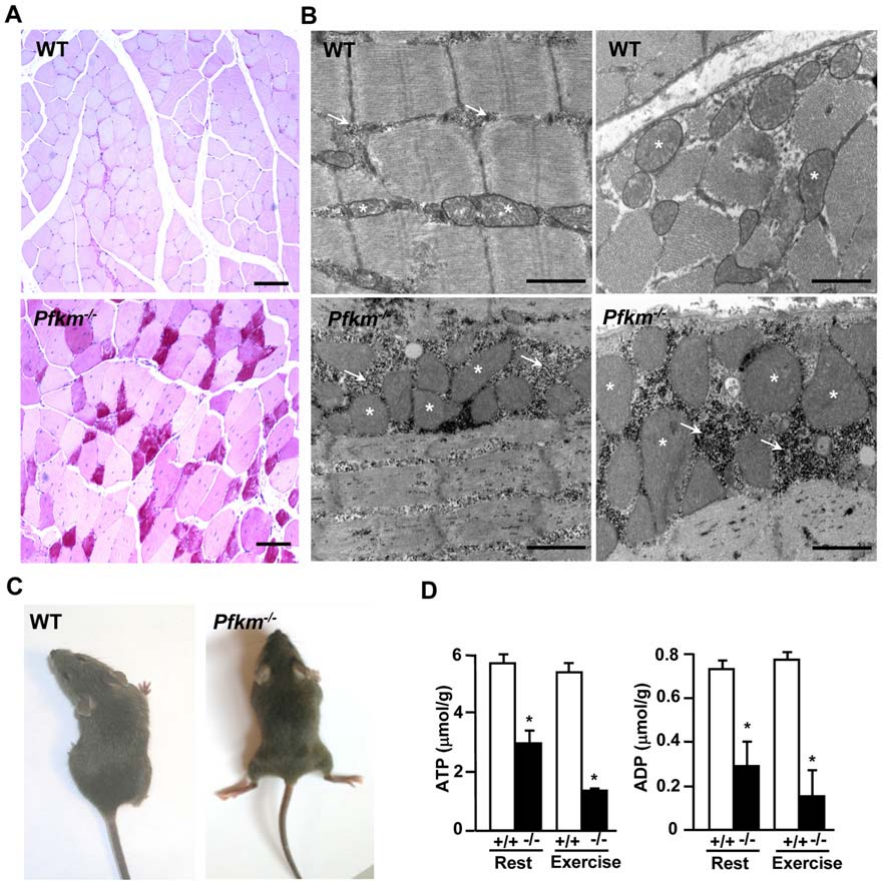
*Pfkm^−/−^* mice develop skeletal muscle glycogenosis and exercise intolerance. (A) Glycogen storage evidenced by PAS staining in skeletal muscle sections from wild-type (WT) and *Pfkm^−/−^* mice. Scale bar 50 µm. (B) Transmission electron microscopic analysis of skeletal muscle. Arrows show glycogen storage and asterisks point to mitochondria. Scale bar 1 µm. (C) *Pfkm^−/−^ mice* showing severe muscle cramps after exercise (5 min). (D) ATP and ADP content was determined in perchloric extracts of skeletal muscle from wild-type (+/+) and and *Pfkm^−/−^* (−/−) mice, in rest and after exercise (5 min), as described in Materials and Methods. Results are mean±SEM of five mice per group. *P<0.05 *vs.* wild-type.

*Pfkm^+/−^* mice showed 50% lower muscle *Pfkm* expression and activity (Figure 1C and 1D). However, this lower enzyme activity in *Pfkm^+/−^* mice did not alter any metabolic parameter, such as glucose-6-phosphate and glycogen levels (data not shown), indicating that half of normal PFK activity is sufficient to prevent metabolic alterations, as observed in heterozygous humans [12]. No *Pfkm* mRNA transcript was observed in skeletal muscle of *Pfkm^−/−^* mice (Figure 1C), in agreement with the lack of enzyme activity (Figure 1D). This deficiency led to increased glucose-6-phosphate (Figure 1E), intracellular glucose (Figure 1F) and glycogen (Figure 1G) content in skeletal muscle. Considerable glycogen storage was also evidenced by histochemical analysis of *Pfkm^−/−^* skeletal muscle (Figure 2A). Furthermore, electron microscopy revealed very high subsarcolemmal and intermyofibrillar accumulation of glycogen, which altered fiber morphology (Figure 2B). In addition, *Pfkm^−/−^* mice showed lower serum lactate levels (Figure 1H), suggesting lower flux through glycolysis in skeletal muscle. Nevertheless, these mice were normoglycemic (*Pfkm^+/+^*, 115±12 *vs. Pfkm^−/−^*, 113±16 mg/dl; (n = 12)). Consistent with this, PFK activity and glucose metabolism were unchanged in the liver (data not shown).

### PFKM–deficient mice show exercise intolerance

Similar to patients with the classical form of GSDVII, two-month-old *Pfkm-*null mice were intolerant to exercise. These mice were unable to run for more than 1.5 min. in a treadmill before developing severe muscle cramps, mainly in the rear limbs (Figure 2C). When exercised, *Pfkm* ^−/−^ mice accumulated higher levels of glucose-6-phosphate (Figure 1E), consistent with increased muscle glucose uptake (Figure 1F) and mobilization of muscle glycogen (Figure 1G).

Since the muscles of these mice fail to perform glycolysis, lactate did not rise after exercise (Figure 1H). Furthermore, in *Pfkm* ^−/−^ skeletal muscles, ATP and ADP levels were lower even in the resting state and fell with exercise (Figure 2D). These lower levels of ATP agreed with the presence of muscle cramps after exercise, spontaneous cramps during manipulation, and immediate *rigor mortis* after death (not shown). Thus, skeletal muscle of *Pfkm* ^−/−^ mice was unable to meet the energy demand required to maintain normal contractile activity.

**Figure 3:**
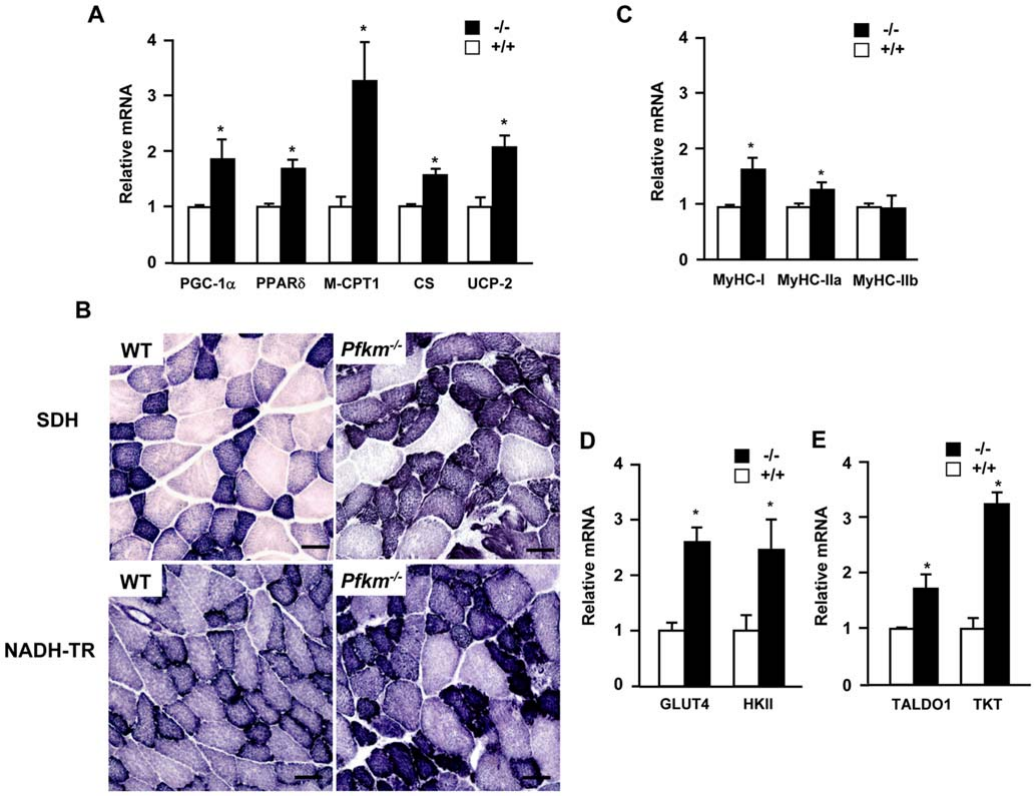
Effects of PFKM deficiency in skeletal muscle markers. (A) Expression of key genes in oxidative metabolism in skeletal muscle of wild-type and *Pfkm^−/−^* mice: Peroxisome proliferator-activated receptor γ coactivator-1α (PGC-1α), peroxisome proliferator-activated receptor δ (PPARδ), carnitine palmytoiltransferase-1 (M-CPT-1), citrate sinthase (CS) and uncoupling protein 2 (UCP-2). (B) Histochemical staining for succinate dehydrogenase (SDH) and NADH-tetrazolium reductase (NADH-TR) activities in skeletal muscle of wild-type and *Pfkm^−/−^* mice. Scale bar 25 µm. (C) Expression of myosin heavy chains in skeletal muscle of wild-type and *Pfkm^−/−^* mice: Type I, IIa ,and IIb myosin heavy chains (MyHC-I, MyHC-IIa, MyHC-IIb). (D) Expression of the key genes in skeletal muscle glucose uptake, glucose transporter 4 (GLUT4) and hexokinase-II (HKII), in wild-type and *Pfkm^−/−^* mice. (E) Expression of pentose phosphate pathway genes, transaldolase (TALDO1) and transketolase (TK), in skeletal muscle of wild-type and *Pfkm^−/−^* mice. Relative expression in A, C, D and E was determined by quantitative PCR analysis of total RNA from skeletal muscle, as indicated in Materials and Methods. Results are mean±SEM of five mice per group. *P<0.05 *vs.* wild-type.

Despite low ATP levels in *Pfkm* ^−/−^ mice, the expression of key genes in oxidative metabolism and mitochondrial biogenesis was higher than in wild-type mice, such as peroxisome proliferator-activated receptor γ coactivator-1α (PGC-1α), peroxisome proliferator-activated receptor δ (PPARδ) muscle carnitine palmitoyltransferase 1 (M-CPT-1), citrate synthase (CS) and uncoupling protein 2 (UCP2) (Figure 3A). Moreover, succinate dehydrogenase and NADH-tetrazolium reductase activities, markers of oxidative capacity, were also higher (Figure 3B). Up-regulation of the expression of type I and IIa myosin heavy chain (MyHC-I and IIa) oxidative-type fiber proteins, without changes in the glycolytic MyHC-IIb, was also observed (Figure 3C). Consistent with these findings, *Pfkm* ^−/−^ mice showed proliferation of enlarged mitochondria surrounded by glycogen depots (Figure 2B). Increased expression of genes involved in glucose uptake and phosphorylation, glucose transporter 4 (GLUT4) and hexokinase-II (HK), was found in skeletal muscle of *Pfkm* ^−/−^ mice (Figure 3D), which also agreed with increased muscle glucose and glucose-6-phosphate content (Figure 1E and 1F). In addition, the expression of the pentose phosphate pathway transaldolase (TALDO1) and transketolase (TK) genes was higher in skeletal muscle of *Pfkm* ^−/−^ than in wild-type mice (Figure 3E). Therefore, despite an increased compensatory response, oxidative metabolism was unable to overcome the glycolysis blockade in *Pfkm* ^−/−^ mice.

### Lack of PFKM alters respiratory muscles and heart

**Figure 4:**
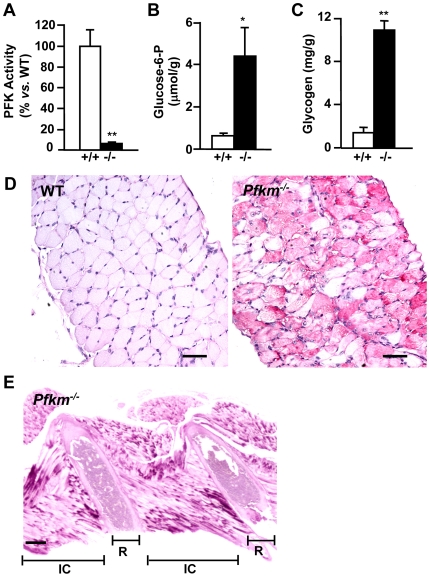
Effect of *Pfkm* ablation in diaphragm glucose metabolism and in respiratory muscle glycogen storage. (A) PFK activity was determined in diaphragm extracts as indicated in Materials and Methods. Basal PFK activity in wild-type mice was 26.5±4.2 U/g tissue. (B,C) Glucose-6-phosphate (B) and glycogen concentrations (C) were determined in diaphragm perchloric extracts from wild-type (+/+) and *Pfkm^−/−^* (−/−) mice, as indicated in Materials and Methods. Results are mean±SEM of five mice per group. *P<0.05, **P<0.01 *vs.* wild-type. (D,E) Glycogen storage evidenced by PAS staining in diaphragm sections (D) from wild-type (wt) and *Pfkm^−/−^* mice (scale bar 50 µm) and in intercostal muscle sections (E) from *Pfkm^−/−^* mice (scale bar 300 µm). IC, intercostal muscles; R, rib.

Respiratory skeletal muscles were also severely altered in *Pfkm* ^−/−^ mice. The lack of PFK activity in diaphragm, led to increased glucose-6-phosphate and glycogen content (Figure 4A–4C). High accumulation of glycogen was also observed in diaphragm (Figure 4D) and intercostal muscle (Figure 4E) sections by PAS staining. These metabolic alterations may have contributed to alter the respiratory capacity of *Pfkm* ^−/−^ mice.

**Figure 5:**
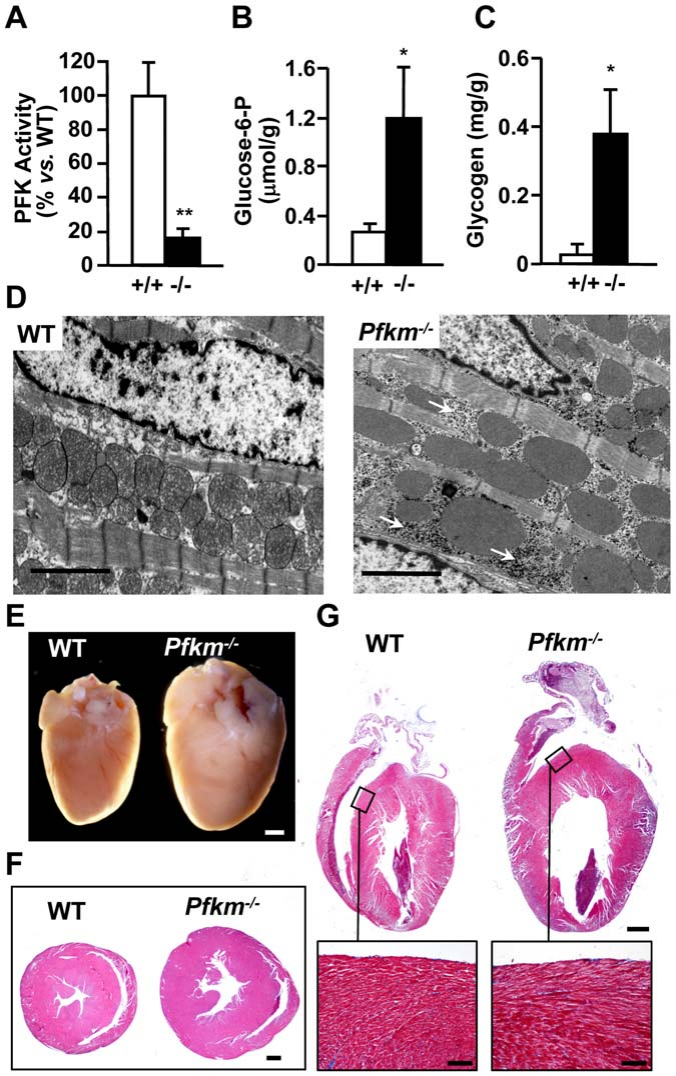
*Pfkm^−/−^* mice show altered heart glucose metabolism and develop cardiomegaly with age. (A) PFK activity was determined in heart extracts. Basal PFK activity in wild-type mice was 27.4±5.4 U/g tissue. (B,C) Glucose-6-phosphate (B) and glycogen concentrations (C) were determined in heart perchloric extracts from 2-month-old wild-type (+/+) and *Pfkm^−/−^* (−/−) mice. Results are mean±SEM of five mice per group. *P<0.05, **P<0.01 *vs.* wild-type. (D) Transmission electron microscopic analysis of cardiac muscle. Arrows show glycogen storage. Scale bar 2 µm. (E,F) One-year-old *Pfkm^−/−^* mice develop cardiac hypertrophy, evidenced by hematoxilin-eosin staining of heart sections (scale bar 1 mm) (E) and cardiomegaly (F). (G) Longitudinal sections of heart from 3-month-old mice stained with Masson trichromic reagent (scale bar 1 mm). Inset shows septum sections (scale bar 50 µm).

Cardiac muscle, which expresses the PFKM, PFKL and PFKP [3], showed lower PFK activity in *Pfkm* ^−/−^ mice (about 20% of wild-type) and higher glucose-6-phosphate and glycogen levels (Figure 5A–5C). In addition, increased glycogen storage was also evident in electron microscopy sections of cardiac muscle (Figure 5D). Two-month-old *Pfkm* ^−/−^ mice showed increased (about 55%) heart weight (*Pfkm^+/+^*, 4.4±0.1 mg/g b.w. *vs. Pfkm^−/−^*, 6.9±0.3 mg/g b.w.; (n = 5) *p<0.01*) and developed cardiac hypertrophy and evident cardiomegaly with age (Figure 5E and 5F). Moreover, left ventricle enlargement without interstitial fibrosis was observed after Masson trichromic staining of longitudinal sections of *Pfkm* ^−/−^ hearts (Figure 5G).

#### *Pfkm^−/−^* mice develop hemolysis, reticulocytosis and splenomegaly

**Figure 6:**
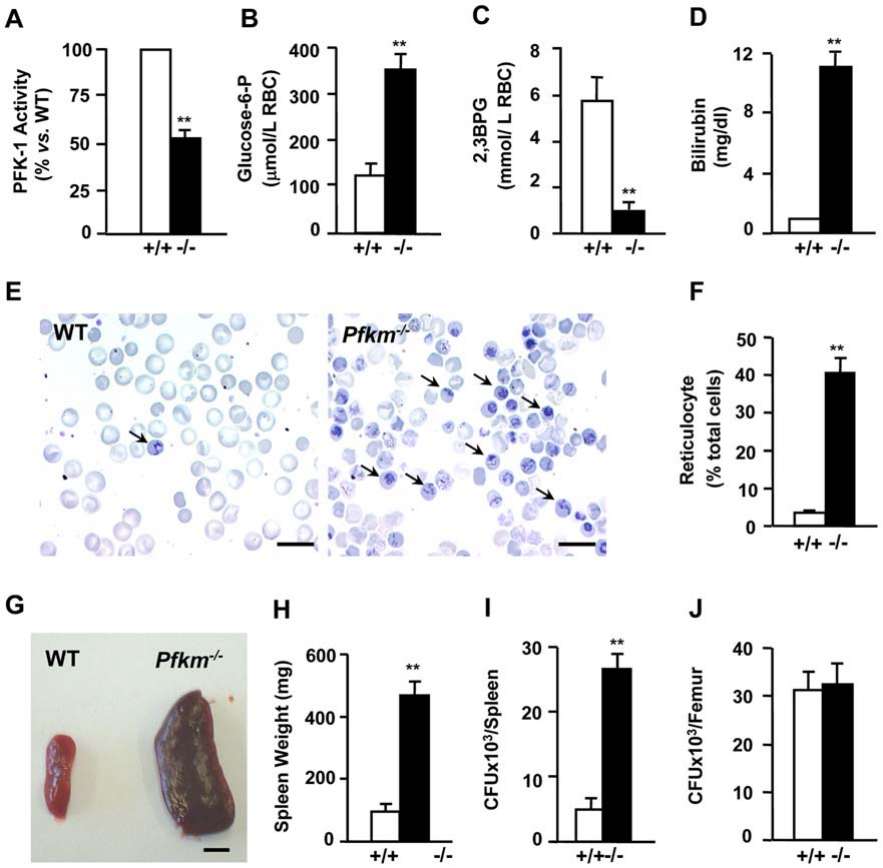
Reduction of erythrocyte PFK activity leads to hemolysis, reticulocytosis, and splenomegaly. (A) PFK activity was determined in blood cell lysates from wild-type (+/+) and *Pfkm^−/−^* (−/−) mice as indicated in Materials and Methods. (B,C) Glucose-6-phosphate (B) and 2,3-bisphosphoglycerate (2,3-BPG) (C) concentrations were determined in blood cell perchloric extracts as indicated in Materials and Methods. (D–F) *Pfkm^−/−^* show high serum bilirubin levels (D) and reticulocyte number (E,F). New methylene blue stained blood samples were extended on slices (E) and counted (F). Arrows indicate reticulocytes. Scale bar 15 µm. (G,H) Splenomegaly in *Pfkm^−/−^* mice. A high increase in spleen size (G) and weight (H) was observed. Scale bar 5 mm. (I,J) Hematopoietic precursors in cultured cells from spleen (I) and femur (J) from wild-type (+/+) and *Pfkm^−/−^* (−/−) mice. Results are mean±SEM of five to eight mice per group. **P<0.01 *vs.* wild-type.

Erythrocytes express both PFKM and PFKL isoenzymes [13]. Consistent with the lack of PFKM in *Pfkm* ^−/−^ mice, erythrocytes showed 50% lower PFK activity and accompanying glucose-6-phosphate accumulation (Figure 6A and 6B). This correlated with lower 2,3-bisphosphoglycerate (2,3-BPG) levels (Figure 6C). These metabolic alterations resulted in increased osmotic fragility of erythrocytes (data not shown) and severe hemolysis. Thus, *Pfkm* ^−/−^ mice had very high levels of serum bilirubin (Figure 6D) and lactate dehydrogenase and lower hematocrit (data not shown). As a consequence, *Pfkm* ^−/−^ mice showed compensatory reticulocytosis (Figure 6E and 6F) and splenomegaly (Figure 6G and 6H), which correlated to increased hematopoietic precursors from spleen, but not bone marrow (Figure 6I and 6J). These alterations are features of GSDVII, in which patients show compensated hemolytic anemia with increased serum bilirubin and reticulocyte count [14].

### Skeletal muscle of *Pfkm* ^−/−^ mice presents increased vascularization and fiber necrosis and regeneration

**Figure 7:**
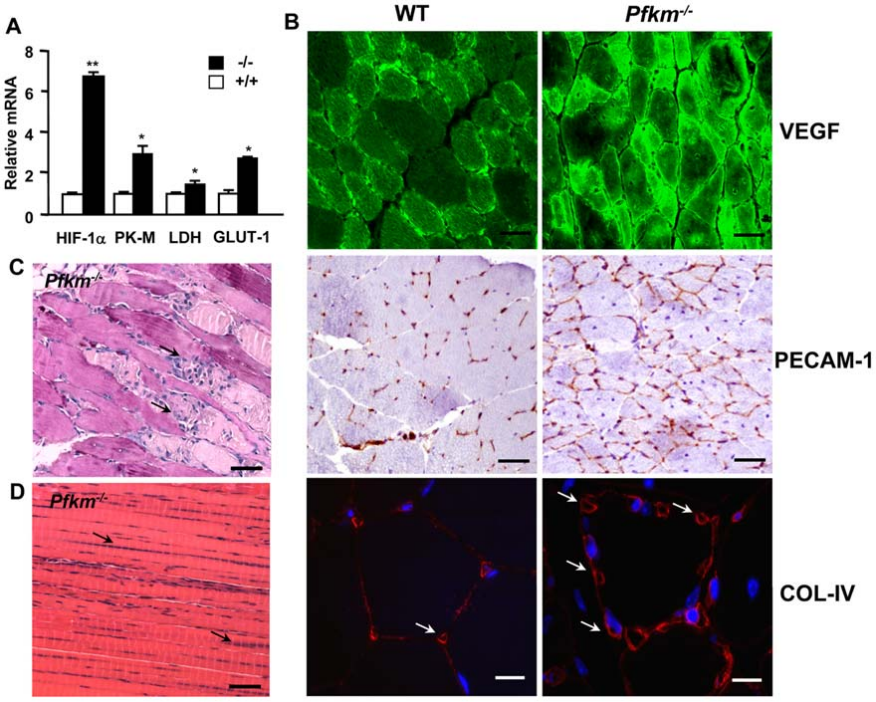
*Pfkm^−/−^* mice show increased skeletal muscle hypoxic markers, vascularization, and fiber necrosis. (A) The expression of the hypoxia-induced factor (HIF-1α), pyruvate kinase (PK-M), lactate dehydrogenase (LDH), and glucose transporter-1 (GLUT-1) in skeletal muscle of *Pfkm^−/−^* mice was determined by quantitative PCR analysis, as indicated in Materials and Methods. Results are mean±SEM of four mice per group. *P<0.05, **P<0.01 *vs.* wild-type. (B) Skeletal muscle sections showed increased immunostaining for VEGF, leading to hypervascularization, as evidenced by greater immunostaining for endothelial cell marker PECAM-1 (scale bar 25 µm) and collagen IV (scale bar 10 µm). Arrows show blood vessels around muscle fiber. (C) Fiber necrosis in skeletal muscle sections of *Pfkm^−/−^* mice. Arrows indicate cell infiltration of necrotic fibers (scale bar 25 µm). (D) Muscle fiber regeneration is evidenced by multiple centrally located nuclei (arrows) (scale bar 25 µm).

The decrease of erythrocyte 2,3-BPG levels increases hemoglobin affinity for oxygen and thus impairs oxygen extraction from hemoglobin [15]. Thus, the inability of oxidative metabolism to compensate for glycolysis blockade in *Pfkm^−/−^* skeletal muscle may also be due to decreased availability of oxygen to generate sufficient energy. Furthermore, consistent with decreased oxygen availability and marked hemolysis, skeletal muscle of *Pfkm^−/−^* mice showed hypoxia, evidenced by higher expression (6-fold) of the hypoxia induced factor 1α (HIF-1 α) (Figure 7A). Moreover, expression of genes activated by HIF-1α, such as pyruvate kinase M (PK-M), lactate dehydrogenase (LDH), and glucose transporter-1 (GLUT1), were up-regulated in this tissue (Figure 7A). This increase in GLUT1 was also consistent with the observed higher intracellular glucose (Figure 1F). The increase in HIF-1α was also parallel to increased vascular endothelial growth factor (VEGF) expression (Figure 7B). In addition, it has been described in skeletal muscle that PGC1α is induced by a lack of oxygen and that PGC1α powerfully regulates VEGF expression [16], which may have also occurred in *Pfkm^−/−^* mice. The increase in VEGF led to hypervascularization, as evidenced by greater immunostaining of the platelet endothelial cell adhesion molecule (PECAM-1), an endothelial cell marker, and collagen IV, a basement membrane marker (Figure 7B). Furthermore, the chronic lower levels of ATP in skeletal muscle of *Pfkm^−/−^* mice resulted in multiple sites of muscle fiber degeneration and necrosis, characterized by inflammatory infiltration of mononucleated cells and by phagocytosis of necrotic fibers (Figure 7C). In addition, intense skeletal muscle regenerative activity was evidenced by wide distribution of centrally-located nuclei fibers in *Pfkm^−/−^* mice (Figure 7D). Thus, severe muscle fiber alterations, in addition to glycogen accumulation, result from PFKM deficiency.

## Discussion

In this study we show that mice with targeted ablation of the muscle isoform of PFK develop myopathic and hemolytic features similar to those noted in type VII glycogenosis in humans. The early lethality observed in *Pfkm^−/−^* mice also resembled the most severe variant of the disease, which presents in infancy and rapidly proceeds to a progressive myopathy and death [6]. Importantly, the full range of phenotypic changes we have observed in our model may impact on diagnosis and detection of human patients since phenotypic heterogeneity is common. In addition, future treatment strategies will need to consider the full extent of pathogenesis to optimize effectivity and safety.

The increased glycogen and glucose-6-phosphate in skeletal muscle observed in *Pfkm^−/−^* mice is the classic hallmark described in biopsies of human patients with GSDVII. Suppression of glycolysis impaired the use of glycogen as a fuel leading to increased storage. Moreover, blood glucose cannot be metabolized by the glycolytic pathway causing glucose-6-phosphate accumulation in skeletal muscle. Allosteric activation of glycogen synthase by glucose-6-phosphate may have contributed to increase glycogen storage [17]. Skeletal muscle uses glucose, either blood- or glycogen- derived, as the major fuel during muscular activity. The impairment of the principal catabolic pathway in skeletal muscle of *Pfkm^−/−^* mice led to energetic deprivation, which resulted in failure to perform exercise. Similarly, PFKM deficient patients show severe alterations in muscle bioenergetics leading to muscle weakness and exercise intolerance [18],[19]. Ineffective utilization of glycogen in patients with type V glycogen storage (GSDV) or McArdle's disease also leads to impairment of exercise capability. GSDV results from deficiency of the muscle isoform of glycogen phosphorylase, which leads to blockade of glycogen breakdown and to high glycogen storage in skeletal muscle [20]. However, GSDV patients show exercise tolerance after carbohydrate infusion since they can metabolize circulating glucose because glycolytic flux is preserved [21]. In contrast, in GSDVII patients, glucose infusion induces exertional fatigue attributed to an insulin-mediated decreased availability of blood free fatty acids and ketone bodies [22].

Muscle fibers of *Pfkm^−/−^* mice failed to generate enough ATP to maintain contractile activity, and mice developed muscle cramps early during the exercise test and with manipulation. In addition, even in rested state, *Pfkm* ^−/−^ mice showed low levels of ATP in the skeletal muscle, which is known to lead to muscle weakness and mitochondrial myopathy in other animal models [23],[24]. Physiological situations involving energy deprivation in skeletal muscle, like exercise and fasting, lead to adaptive changes towards the oxidation of fat as a fuel [25]. In skeletal muscle of *Pfkm* ^−/−^ mice, increased expression of oxidative marker genes and proliferation of enlarged mitochondria revealed an attempt to overcome glycolysis deficiency by shifting substrate metabolism toward a higher reliance on oxidative metabolism. Factors involved in this adaptation included PGC-1α, PPARδ and muscle CPT-1, which are responsible for mitochondrial biogenesis, oxidative phosphorylation and fatty acid oxidation [25]. Furthermore, PGC-1α and PPARδ may have been involved in structural changes towards the formation of oxidative muscle fibers by increasing the expression of MyHC-I [26],[27]. Moreover, PGC-1α up-regulation was probably responsible for the increased expression of GLUT-4 and HK-II in skeletal muscle of *Pfkm^−/−^* mice [28]. This led to enhanced glucose uptake and phosphorylation, also consistent with the high levels of glucose and glucose-6-phosphate detected in skeletal muscle of *Pfkm^−/−^* mice. In addition, the increased expression of transaldolase and transketolase enzymes suggested that glucose could be used through the pentose phosphate pathway in skeletal muscle of *Pfkm^−/−^* mice. However, despite these compensatory responses, oxidative metabolism was unable to overcome the glycolysis blockade in *Pfkm* ^−/−^ mice.

Anaplerosis of the tricarboxylic acid (TCA) or Krebs cycle plays a key role in oxidative metabolism in skeletal muscle by providing the TCA cycle with intermediates to permit its continued function. Impaired production of glycolytic substrates could limit oxidative metabolism by reducing concentrations of Krebs cycle intermediates [29],[30]. Blockade of glucose utilization through the glycolysis pathway in skeletal muscle of *Pfkm* ^−/−^ mice may lead to impaired production of the glucose-derived anaplerotic substrates phosphoenolpyruvate and pyruvate. Dysregulation of the TCA cycle intermediates probably impaired oxidative phosphorylation and the ability of skeletal muscle in *Pfkm* ^−/−^ mice to generate an adequate amount of ATP. The significance of the regulation of TCA cycle intermediates in the control of skeletal muscle energy metabolism has clearly been shown in mice overexpressing phosphoenolpyruvate carboxykinase (PEPCK-C). PEPCK-C transgenic mice show increased oxidative capacity in skeletal muscle leading to enhanced exercise performance [31].

GSDVII is also characterized by compensated hemolytic anemia due to reduction in the erythrocyte PFK activity. *Pfkm^−/−^* mice clearly underwent hemolysis and compensatory erythropoiesis evidenced by marked reticulocytosis. Since erythrocytes lack mitochondria, glycolysis is essential for their energy metabolism. Consequently, although erythrocytes of *Pfkm^−/−^* mice preserve about half of the PFK activity observed in wild-type mice, it was not enough to maintain erythrocyte integrity. Moreover, the kinetic properties of residual L homotetramer may turn it somehow dysfunctional in *Pfkm^−/−^* erythrocytes [32]. Removal of defective erythrocytes was probably responsible for the increased spleen size in *Pfkm^−/−^* mice. Splenomegaly has broadly been described as a result of hemolysis or hematopoietic stress in several diseases [33],[34]. Thus, increased hematopoiesis may have also contributed to increase spleen size in *Pfkm^−/−^* mice. Similar hematological features are found in spontaneous mutant mice with reduced activity of the glycolytic enzyme pyruvate kinase (*Pk-1^slc^*) in red blood cells [35].

Lower PFK activity in erythrocytes of *Pfkm^−/−^* mice led to lower concentrations of glycolytic intermediates and 2,3-BPG. In turn, low levels of 2,3-BPG increase the oxygen affinity of hemoglobin, reducing oxygen delivery to the tissues and stimulating erythropoiesis. Skeletal muscle requires large amounts of oxygen during intense exercise and alterations in the affinity of hemoglobin for oxygen could impair muscle performance [15]. Consistent with decreased oxygen availability and marked hemolysis, skeletal muscle of *Pfkm^−/−^* mice showed features of hypoxia and angiogenesis together with necrosis and intense regenerative activity. This decreased oxygen availability probably contributed to impair the compensatory oxidative metabolism in the skeletal muscle of PFK deficient mice, exacerbating its loss of functionality. In addition, changes in oxygen delivery to tissues may result in lower respiratory and cardiac function in *Pfkm^−/−^* mice.

Involvement of respiratory and cardiac muscles in the pathogenesis of GSDVII is not clearly understood. Myopathic alterations in the respiratory muscles are responsible for loss of respiratory function and even death in a wide spectrum of muscle disorders [36],[37] and other glycogen storage diseases [38],[39]. In addition, premature death due to a respiratory failure is a feature of the severe infantile form of GSDVII [6],[40]. The structural and metabolic abnormalities observed in the diaphragm and respiratory muscles of *Pfkm^−/−^* mice suggest impaired respiratory function and may have contributed to the lethality observed in these mice. On the other hand, cardiac abnormalities, such as low voltage electrocardiogram, tachycardia, ventricular hypertrophy and atrium enlargement, have only been described in a few patients [41]. Cardiac hypertrophy may result as an adaptive response to increased workload, and prolonged hypertrophy is associated with increased risk for sudden death or progression to heart failure [42]. Although most frequent causes of heart hypertrophy are chronic hypertension, exercise, myocardial infarction or aortic valve stenosis, several reports point to defects in cardiac energetic metabolism underlying heart enlargement [43]. Thus, heart specific ablation of GLUT-4 glucose transporter or deletion of the adenine nucleotide translocator-1 gene lead to heart hypertrophy in mice [24],[44]. Therefore, altered glucose metabolism in the heart of Pfkm^−/−^ mice may have led to deficient energy production in cardiomyocyte and compensatory chronic heart hypertrophy, which probably increased mortality in these mice. These results suggest that the cardiac pathology in GSDVII may probably be underestimated or misdiagnosed [41]. In addition, this study indicates that symptoms other than classically described may be taken into consideration for the diagnostic of the GSDVII.

In summary, these results indicate that the skeletal and cardiac muscle impairments observed in *Pfkm^−/−^* mice interact with disturbed erythrocyte metabolism to produce the heterogeneous and complex pathology characteristic of type VII glycogen storage disease. The availability of this murine model of GSDVII allows determination of the role of such metabolic alterations in different tissues and organs together with their interactions, and, importantly, allows the study of GSDVII as a systemic disorder, not simply as a muscle glycogenosis. Moreover, *Pfkm^−/−^* mice constitute a unique model of GSDVII, which will most likely be very useful for the design and assessment of new therapeutic interventions for this disease.

## Materials and Methods

### Generation of *Pfkm^−/−^* mice

Genomic clones for mouse *Pfkm* were isolated from a mouse 129/SvJ library (Stratagene). To construct the targeting vector, two fragments of the genomic DNA flanking the exon 3 were subcloned at convenient restriction sites in the pPNT vector. Linearized pPTN/*pfkm* was transfected into 129/SvJ derived embryonic stem cells (ES) (CMTI-1, Speciality Media). Selection was performed with G418 and gancyclovir, and resistant clones were screened for homologous recombination by Southern blot. Targeted ES cells were injected into blastocysts from C57BL/6J mice and transferred into uteri of pseudopregnant females. Chimeric males were mated to C57BL/6J females and the offspring was screened by PCR analysis using both locus-specific and Neo cassette-specific primers: PFK-Fw: 5′-AATGCACTCCGATCTGCTCC-3′; Neo: 5′-CGCCTTCTATCGCCTTCTTG ACGAGTTCTT-3′; PFK-Rev: 5′-GCAAGCAATGCCTAAATCTG-3′. Homozygous mutant mice were obtained by mating heterozygous littermates. Mice were fed *ad libitum* with a standard diet (Panlab, Barcelona, Spain) and maintained under a light-dark cycle of 12 h (lights on at 9:00 A.M.). Animals were killed and samples were taken between 9:00 and 10:00 A.M. In the experiments described, male mice, aged 2–3 months were used with littermates as controls. All experimental procedures involving mice were approved by the Ethics Committee in Animal and Human Experimentation of the Universitat Autònoma de Barcelona.

### RNA analysis

Total RNA was obtained from skeletal muscle samples and analyzed by Northern blot. Northern blots were hybridized to ^32^P-labeled *pfkm* cDNA probe labeled following the method of random oligopriming, as described by the manufacturer (Amersham Corp.). For real-time qPCR, 1 µg of RNA samples was used as a template to synthesize cDNA in a volume of 20 µl (Omniscript kit, Qiagen). Oligo-dT was used as a primer for the reaction in the presence of Protector RNase inhibitor (Roche). RT-PCR was performed in a SmartCycler II (Cepheid) using QuantiTect SYBR Green PCR kit (Qiagen). The sequences of the respective sense and antisense oligonucleotide primers were: Primers sequences: PGC-1α: (F) ATACCGCAAAGAGCACGAGAAG and (R) CTCAAGAGCAGCGAAAGCGTCACAG; PPARδ: (F) TCCATCGTCAACAAAGACGGG and (R) ACTTGGGCTCAATGATGTCAC; M-CPT1: (F) GCACACCAGGCAGTAGCTTT and (R) CAGGAGTTGATTCCAGACAGGTA; CS: (F) TGCCCACACAAGCCATTTG and (R) CTGACACGTCTTTGCCAACTT; HIF-1α: (F) AGCCC TAGATGGCTTTGTGA and (R) TATCGAGGCTGTGTCGACTG; PK-M: (F) CGATCTGTGGAGATGCTGAA and (R) AATGGGATCAGATGCAAAGC; LDH: (F) TGTCTCCAGCAAAGACTACTGT and (R) GACTGTACTTGACAAT GTTGGGA; GLUT-1: (F) CAGTTCGGCTATAACACTGGTG and (R) GCCCCCGACAGAGAAGATG; MyHC-I: (F) AGAGGGTGGCAAAGTCACTG and (R) GCCATGTCCTCGATCTTGTC; MyHC-IIa: (F) CGATGA TCTTGCCAGTAATG and (R) TGATAACTGAGATACCAGCG; MyHC-IIb: (F) ACAGACTAAAGTGAAAGCC and (R) CTCTCAACAGAAAGATGGAT; GLUT4: (F) GACGGACACTCCATCTGTTG and (R) CATAGCTCATGGCTGG AACC; HKII: (F) GAAGGGGCTAGGAGCTACCA and (R) CTCGGAG CACACGGAAGTT; TALDO1: (F) GATTCCAGGCCGTGTATCCAC and (R) AATCCCCTCCCAGGTTGATGA; TKT: (F) TGGCATACACAGGCAAATACTT and (R) TCCAGCTTGTAAATTCCAGCAA and 36B4: (F) GGCCCTGCACTCTCGCTTT and (R) TGCCAGGACGCGCTTGT. Data was normalized with 36B4 gene values and analyzed as previously described [45].

### Enzyme and metabolite assays

To determine PFK activity and the concentration of metabolites mice were anesthetized with a mixture of ketamine (100 mg/kg) and xylacine (10 mg/kg). Afterwards, skeletal muscle was freeze clamped *in situ*, and kept at −80°C until analysis. Diaphragm and heart were rapidly excised, weighed, frozen in liquid nitrogen and kept at −80°C. Heparinized blood samples were centrifuged, cells collected and frozen. For PFK activity, samples were homogenized in 10 volumes (1 ml/100 mg tissue) of an ice-cold buffer (pH 7.4) containing 20 mM Tris-HCl, 100 mM KCl, 5 mM MgCl_2_, 5 mM Phosphate Buffer and 30% Glycerol. Samples were centrifugued and PFK activity was determined in the presence of 6 mM fructose 6-phosphate and 18 mM glucose 6-phosphate by spectrophotometric analysis as previously indicated [46]. The concentrations of glycogen, glucose 6-phosphate, glucose and 2,3-BPG were measured in perchloric extracts, which were adjusted to pH 5 with 5 M K_2_CO_3_ to determine glycogen and glucose, and to pH 7 for glucose 6-phosphate and 2,3-BPG. Glycogen levels were measured using the α-amyloglucosidase method [47]. Glucose and glucose 6-phosphate concentrations were determined enzymatically [48]. 2,3-BPG was determined using a specific kit (Roche Diagnostics GMBH). The concentration of ATP and ADP was determined as described previously [49],[50]. Serum lactate dehydrogenase activity and total bilirubin and lactate levels were measured in the autoanalyzer Pentra400 (ABX Diagnostics) using specific kits (ABX Diagnostics). Glucose concentration in blood was determined by using a Glucometer Elite (Bayer) following the manufacturer's instructions.

### Exercise test

Mice were exercised for 5 min on an enclosed treadmill LE-8708 (Panlab) supplied with an electrified grid at the rear of the belt to provide motivation. The speed of the belt was 30 cm/sec.

### Retyculocyte count and hematopoietic cultures

To determine the number of retyculocytes, blood samples were stained with new methylene blue, extended on slices, and counted under microscope. Hematopoietic cultures were performed in extracts of bone marrow and spleen. Triplicate assays were done in 35 mm plates. Samples were cultured for 7 days at 37°C, 5% CO_2_ and 95% relative humidity in MethoCult GF M3434 medium (StemCell Technologies Inc.). Colonies were counted under inverted microscope including CFU-GM, BFU-E, and CFU-Mix.

### Histochemical analysis

Skeletal muscle and heart were fixed for 12 to 24 h in formalin, embedded in paraffin and sectioned. To determine muscle morphology, sections were stained with hematoxylin/eosin. Glycogen content was analyzed by Periodic Acid Schiff (PAS) staining. Heart fibrosis was determined by Masson trichrome staining. For histochemical analysis of succinate dehydrogenase (SDH) and NADH-tetrazolium reductase (NADH-TR) activities, gastrocnemius muscle was dissected and frozen in isopentane supercooled with liquid nitrogen. Frozen sections were analyzed as previously indicated [51],[52].

### Immunohistochemistry

For immunohistochemical detection of VEGF and collagen IV proteins, paraffin sections were incubated overnight at 4°C with rabbit anti-mouse VEGF antibody (Santa Cruz) diluted at 1∶50 and with rabbit anti-mouse collagen IV antibody (Chemicon Inc.) diluted at 1∶100. For immunohistochemical detection of PECAM-1, cryosections were incubated overnight at 4°C with rat anti-mouse PECAM-1 antibody (Pharmingen BDbiosciences) diluted at 1∶100. Afterwards, samples were incubated with the biotinylated secondary antibodies (dilution 1∶200): Rabbit against rat IgG (Vector laboratories) or goat against rabbit IgG (Vector laboratories). The localization of VEGF was determined using streptavidin conjugate Alexa fluor 488 (Molecular Probes), collagen IV using streptavidin conjugate Alexa fluor 568 (Molecular Probes) and PECAM-1 by ABC peroxidase mouse IgG staining kit (Pierce Biotechnology).

### Transmission electron microscopic analysis

Skeletal and cardiac muscle samples were obtained and fixed in 2.5% glutaraldehyde and 2% paraformaldehyde for 2 h at 4°C. After washing in cold cacodylate buffer, the specimens were postfixed in 1% osmium tetroxide, stained in aqueous uranyl acetate, and then dehydrated through a graded ethanol series and embedded in epoxy resin. Ultrathin sections (600–800 Å) from the resin blocks were stained using lead citrate and examined in a transmission electron microscopy (Hitachi H-7000).

### Statistical analysis

All values were expressed as mean±SEM. Two-tailed P values were calculated by unpaired Student's t test. Differences were considered statistically significant at *P* values less than 0.05.

## References

[1] Vora S (1982). Isozymes of phosphofructokinase.. Isozymes Curr Top Biol Med Res.

[2] Vora S, Seaman C, Durham S, Piomelli S (1980). Isozymes of human phosphofructokinase: identification and subunit structural characterization of a new system.. Proc Natl Acad Sci U S A.

[3] Dunaway GA, Kasten TP (1987). Nature of the subunits of the 6-phosphofructo-1-kinase isoenzymes from rat tissues.. Biochem J.

[4] Tarui S, Okuno G, Ikura Y, Tanaka T, Suda M (1965). Phosphofructokinase deficiency in skeletal muscle. A new type of glycogenosis.. Biochem Biophys Res Commun.

[5] Raben N, Sherman JB (1995). Mutations in muscle phosphofructokinase gene.. Hum Mutat.

[6] Servidei S, Bonilla E, Diedrich RG, Kornfeld M, Oates JD (1986). Fatal infantile form of muscle phosphofructokinase deficiency.. Neurology.

[7] Amit R, Bashan N, Abarbanel JM, Shapira Y, Sofer S (1992). Fatal Familial Infantile Glycogen-Storage-Disease - Multisystem Phosphofructokinase Deficiency.. Muscle & Nerve.

[8] Stollberger C, Finsterer J, Bittner RE (1997). Angina for 14 years.. Lancet.

[9] Giger U, Harvey JW, Yamaguchi RA, McNulty PK, Chiapella A (1985). Inherited phosphofructokinase deficiency in dogs with hyperventilation-induced hemolysis: increased in vitro and in vivo alkaline fragility of erythrocytes.. Blood.

[10] Giger U, Smith BF, Woods CB, Patterson DF, Stedman H (1992). Inherited phosphofructokinase deficiency in an American cocker spaniel.. J Am Vet Med Assoc.

[11] Vora S, Giger U, Turchen S, Harvey JW (1985). Characterization of the enzymatic lesion in inherited phosphofructokinase deficiency in the dog: an animal analogue of human glycogen storage disease type VII.. Proc Natl Acad Sci U S A.

[12] Vorgerd M, Karitzky J, Ristow M, Van Schaftingen E, Tegenthoff M (1996). Muscle phosphofructokinase deficiency in two generations.. J Neurol Sci.

[13] Ronquist G, Rudolphi O, Engstrom I, Waldenstrom A (2001). Familial phosphofructokinase deficiency is associated with a disturbed calcium homeostasis in erythrocytes.. J Intern Med.

[14] Nakajima H, Raben N, Hamaguchi T, Yamasaki T (2002). Phosphofructokinase deficiency; past, present and future.. Curr Mol Med.

[15] McCully K, Chance B, Giger U (1999). In vivo determination of altered hemoglobin saturation in dogs with M-type phosphofructokinase deficiency.. Muscle Nerve.

[16] Arany Z, Foo SY, Ma Y, Ruas JL, Bommi-Reddy A (2008). HIF-independent regulation of VEGF and angiogenesis by the transcriptional coactivator PGC-1alpha.. Nature.

[17] Villar-Palasi C, Guinovart JJ (1997). The role of glucose 6-phosphate in the control of glycogen synthase.. FASEB J.

[18] Bertocci LA, Haller RG, Lewis SF, Fleckenstein JL, Nunnally RL (1991). Abnormal high-energy phosphate metabolism in human muscle phosphofructokinase deficiency.. J Appl Physiol.

[19] Argov Z, Bank WJ, Maris J, Leigh JS, Chance B (1987). Muscle energy metabolism in human phosphofructokinase deficiency as recorded by 31P nuclear magnetic resonance spectroscopy.. Ann Neurol.

[20] Dimaur S, Andreu AL, Bruno C, Hadjigeorgiou GM (2002). Myophosphorylase deficiency (glycogenosis type V; McArdle disease).. Curr Mol Med.

[21] Haller RG, Vissing J (2002). Spontaneous “second wind” and glucose-induced second “second wind” in McArdle disease: oxidative mechanisms.. Arch Neurol.

[22] Haller RG, Lewis SF (1991). Glucose-induced exertional fatigue in muscle phosphofructokinase deficiency.. N Engl J Med.

[23] Han DH, Nolte LA, Ju JS, Coleman T, Holloszy JO (2004). UCP-mediated energy depletion in skeletal muscle increases glucose transport despite lipid accumulation and mitochondrial dysfunction.. Am J Physiol Endocrinol Metab.

[24] Graham BH, Waymire KG, Cottrell B, Trounce IA, MacGregor GR (1997). A mouse model for mitochondrial myopathy and cardiomyopathy resulting from a deficiency in the heart/muscle isoform of the adenine nucleotide translocator.. Nat Genet.

[25] de Lange P, Moreno M, Silvestri E, Lombardi A, Goglia F (2007). Fuel economy in food-deprived skeletal muscle: signaling pathways and regulatory mechanisms.. FASEB J.

[26] Wang YX, Zhang CL, Yu RT, Cho HK, Nelson MC (2004). Regulation of muscle fiber type and running endurance by PPARdelta.. PLoS Biol.

[27] Liang H, Ward WF (2006). PGC-1alpha: a key regulator of energy metabolism.. Adv Physiol Educ.

[28] Wende AR, Schaeffer PJ, Parker GJ, Zechner C, Han DH (2007). A role for the transcriptional coactivator PGC-1alpha in muscle refueling.. J Biol Chem.

[29] Gibala MJ, Young ME, Taegtmeyer H (2000). Anaplerosis of the citric acid cycle: role in energy metabolism of heart and skeletal muscle.. Acta Physiol Scand.

[30] Owen OE, Kalhan SC, Hanson RW (2002). The key role of anaplerosis and cataplerosis for citric acid cycle function.. J Biol Chem.

[31] Hakimi P, Yang J, Casadesus G, Massillon D, Tolentino-Silva F (2007). Overexpression of the cytosolic form of phosphoenolpyruvate carboxykinase (GTP) in skeletal muscle repatterns energy metabolism in the mouse.. J Biol Chem.

[32] Vora S, Davidson M, Seaman C, Miranda AF, Noble NA (1983). Heterogeneity of the molecular lesions in inherited phosphofructokinase deficiency.. J Clin Invest.

[33] Aessopos A, Farmakis D, Deftereos S, Tsironi M, Polonifi A (2005). Cardiovascular effects of splenomegaly and splenectomy in beta-thalassemia.. Ann Hematol.

[34] Lenox LE, Perry JM, Paulson RF (2005). BMP4 and Madh5 regulate the erythroid response to acute anemia.. Blood.

[35] Morimoto M, Kanno H, Asai H, Tsujimura T, Fujii H (1995). Pyruvate kinase deficiency of mice associated with nonspherocytic hemolytic anemia and cure of the anemia by marrow transplantation without host irradiation.. Blood.

[36] Lynn DJ, Woda RP, Mendell JR (1994). Respiratory dysfunction in muscular dystrophy and other myopathies.. Clin Chest Med.

[37] Shahrizaila N, Kinnear WJ, Wills AJ (2006). Respiratory involvement in inherited primary muscle conditions.. J Neurol Neurosurg Psychiatry.

[38] Milstein JM, Herron TM, Haas JE (1989). Fatal infantile muscle phosphorylase deficiency.. J Child Neurol.

[39] Pellegrini N, Laforet P, Orlikowski D, Pellegrini M, Caillaud C (2005). Respiratory insufficiency and limb muscle weakness in adults with Pompe's disease.. Eur Respir J.

[40] Guibaud P, Carrier H, Mathieu M, Dorche C, Parchoux B (1978). [Familial congenital muscular dystrophy caused by phosphofructokinase deficiency].. Arch Fr Pediatr.

[41] Finsterer J, Stollberger C, Kopsa W (2002). Neurologic and cardiac progression of glycogenosis type VII over an eight-year period.. South Med J.

[42] Frey N, Olson EN (2003). Cardiac hypertrophy: the good, the bad, and the ugly.. Annu Rev Physiol.

[43] Ashrafian H, Redwood C, Blair E, Watkins H (2003). Hypertrophic cardiomyopathy: a paradigm for myocardial energy depletion.. Trends Genet.

[44] Abel ED, Kaulbach HC, Tian R, Hopkins JC, Duffy J (1999). Cardiac hypertrophy with preserved contractile function after selective deletion of GLUT4 from the heart.. J Clin Invest.

[45] Pfaffl MW (2001). A new mathematical model for relative quantification in real-time RT-PCR.. Nucleic Acids Res.

[46] Castano JG, Nieto A, Feliu JE (1979). Inactivation of phosphofructokinase by glucagon in rat hepatocytes.. J Biol Chem.

[47] Keppler D, Decker K, Bergmeyer HU (1981). Glycogen.. Methods of Enzymatic Analysis.

[48] Michal G, Bergmeyer HU (1981). Glucose 6-Phosphate.. Methods of Enzymatic Analysis.

[49] Lambrecht M, Transtschold D, Bergmeyer HU (1965). ATP determination with hexokinase and glucose-6-phosphate dehidrogenase.. Methods of Enzymatic Analysis.

[50] Adam H, Bergmeyer HU (1965). Determination of adenosine-5′ -diphosphate and adenosine-5′ -monophosphate.. Methods of Enzymatic Analysis.

[51] Nachlas MM, Tsou KC, De Souza E, Cheng CS, Seligman AM (1957). Cytochemical demonstration of succinic dehydrogenase by the use of a new p-nitrophenyl substituted ditetrazole.. J Histochem Cytochem.

[52] Dubowitz V, Brooke MH (1973). Muscle biopsy—a modern approach.

